# Time-course, negative-stain electron microscopy–based analysis for investigating protein–protein interactions at the single-molecule level

**DOI:** 10.1074/jbc.M117.808352

**Published:** 2017-09-29

**Authors:** Bartek Nogal, Charles A. Bowman, Andrew B. Ward

**Affiliations:** From the Department of Integrative Structural and Computational Biology, The Scripps Research Institute, La Jolla, California 92037

**Keywords:** electron microscopy (EM), glycoprotein, human immunodeficiency virus (HIV), kinetics, protein-protein interaction, broadly neutralizing antibodies, single-particle analysis

## Abstract

Several biophysical approaches are available to study protein–protein interactions. Most approaches are conducted in bulk solution, and are therefore limited to an average measurement of the ensemble of molecular interactions. Here, we show how single-particle EM can enrich our understanding of protein–protein interactions at the single-molecule level and potentially capture states that are unobservable with ensemble methods because they are below the limit of detection or not conducted on an appropriate time scale. Using the HIV-1 envelope glycoprotein (Env) and its interaction with receptor CD4-binding site neutralizing antibodies as a model system, we both corroborate ensemble kinetics-derived parameters and demonstrate how time-course EM can further dissect stoichiometric states of complexes that are not readily observable with other methods. Visualization of the kinetics and stoichiometry of Env–antibody complexes demonstrated the applicability of our approach to qualitatively and semi-quantitatively differentiate two highly similar neutralizing antibodies. Furthermore, implementation of machine-learning techniques for sorting class averages of these complexes into discrete subclasses of particles helped reduce human bias. Our data provide proof of concept that single-particle EM can be used to generate a “visual” kinetic profile that should be amenable to studying many other protein–protein interactions, is relatively simple and complementary to well-established biophysical approaches. Moreover, our method provides critical insights into broadly neutralizing antibody recognition of Env, which may inform vaccine immunogen design and immunotherapeutic development.

## Introduction

Binding parameters describing protein–protein interactions can be derived via different methodologies. These include well-established approaches such as ELISAs, SPR, isothermal calorimetry (ITC),^2^ and biolayer interferometry (BLI). ELISA, SPR, and BLI all utilize surface immobilization of proteins, whereas ITC measures interactions of free molecules in solution, giving each of the methods a unique set of advantages and potential drawbacks (Table 1). When combined, these techniques provide complementary biophysical measurements to create a highly detailed understanding of protein–protein interactions. ITC generates thermodynamically-derived, solution-based stoichiometry and affinity. The immobilization-based SPR and BLI supply estimates of the kinetic parameters *k*_on_ and *k*_off_, as well as estimates of the affinity and stoichiometry (S_m_) (in the case of SPR) of the immobilized ligand. Moreover, although SPR can be done in high-throughput 96- or 384-well format with dense 2D surface arrays, the necessary modification for the purpose of chip immobilization may negatively impact the protein (1, 2). Solution-based ITC, on the other hand, is low throughput and requires large quantities of material. In some cases the binding affinity measurements can vary substantially between techniques, as was recently reported with anti-HIV antibodies (2). Here, we observed that the ITC-derived PGV04 *K_D_* was 20-fold higher than that obtained with SPR. We attributed this discrepancy between the measurements to several aspects of the two techniques. These included the uncertainty in kinetic modeling necessary in SPR (particularly due to the negligible off-rates), conformational changes due to ligand immobilization, as well as the fact that ITC-measured binding event induced entropy changes would differ between a free *versus* immobilized ligand. However, for the purpose of evaluating and/or comparing protein-protein interactions, all the techniques are likely to provide similar qualitative answers and the choice of technique may be a matter of matching it with the intended application. For example, if tens of samples are to be screened in short order, the choice of a technique other than BLI (or SPR) may be precluded as its 384-well plate format lends itself to the highest throughput and thus is an ideal screening tool.

**Table 1:** **Commonly used kinetics measurement techniques and the associated biophysical measurements**

	Kinetic measurements	Typical time allowed for ligand-analyte association	Description
**Surface plasmon resonance (SPR)**	*K_D_*, *k*_on_, *k*_off_, S_m_	∼300 s	Kinetics measurements are inferred from measuring surface binding event-induced changes in optical parameters (increase in refractive index) due to immobilized ligand-analyte interaction within a chip flow cell
**Biolayer interferometry (BLI)**	*K_D_*, *k*_on_, *k*_off_	∼300 s	Kinetics measurements are inferred from measuring surface binding event-induced changes in optical parameters (wavelength of reflected light) due to immobilized ligand-analyte interaction on 96-well based biosensor tips
**Isothermal calorimetry (ITC)**	S, *K_D_*	∼1 h	Heat generated/absorbed from ligand-analyte interactions in solution is directly measured during gradual titration, then precise measures of affinity, molar ratios, enthalpy, entropy and heat capacity are derived
**ELISA**	*K_D_* (estimate)	>1 h	Multiple approaches possible, with affinity estimated at half-maximal binding using non-linear curve fitting or Scatchard plot
**Fluorescence resonance energy transfer (FRET)**	*K_D_*, *k*_off_	Nanoseconds to hours	Competitive binding assays are used where a labeled ligand is bound and later displaced by a competitive inhibitor; affinity is derived by measuring the inhibition constant *K_i_*; structural information such as binding distances may also be inferred

We endeavored to evaluate single particle electron microscopy (EM) methods as an additional tool to study protein–protein interactions. Visual examination of single protein complexes provides direct observations complementary to ensemble kinetics methodologies. These observations include the ability to observe intermediate, pre-equilibrium states (either conformational or stoichiometric), observation of minor subpopulations of particles that may give insight into binding mechanisms and/or previously unknown interactions, and in the case of negative-stain electron microscopy (NS-EM), moderate throughput. As a model system we have studied the interaction between HIV Env glycoprotein (Env) and broadly neutralizing antibodies (bnAbs), specifically those directed at the CD4 receptor-binding site. bnAbs recognize conserved epitopes on the highly variable and heavily glycosylated trimeric Env on the surface of HIV (3). These bnAbs, isolated from chronically infected patients provide evidence that the human immune system is capable of generating effective immune responses against HIV and give hope that an effective HIV vaccine can be achieved. By harnessing a structural and biophysical understanding of how these bnAbs interact with Env, researchers are engineering a next generation of protein subunit vaccines designed to elicit these protective bnAbs in naive individuals. Some of the best-characterized and most potent bnAbs, chosen as part of this proof-of-concept study, are also being investigated as therapeutic vaccines (4–9).

The binding of various subclasses of bnAbs to Env, particularly the soluble BG505 SOSIP.664 trimer, have been relatively well-characterized (2, 10–14). The prototypic bnAb has a modest on-rate, low off-rate, high-affinity and binding stoichiometry. These properties correlate well with potent virus neutralization (2). In addition to these data, there are a large number of structures of these bnAbs in complex with the Env trimer, and single-particle EM has made increasing contributions in this regard (11–13, 15–19). In fact, the rationale for this study was based on the observation that some trimer–bnAb complexes could be sorted into subclasses with different stoichiometries of antibodies bound. For example, in a single particle cryo-electron microscopy (cryoEM) study Lyumkis *et al.* (11) observed that ∼44% of the BG505 SOSIP.664 trimers were bound by three CD4-binding site (CD4bs)-directed PGV04 fragments antigen binding (Fabs), with smaller proportions being attributed to one, two, or no Fab-bound classes, despite the presence of severalfold molar excess PGV04. When the stoichiometry was measured using ITC, the calculated value was two Fabs per trimer. Hence, the EM data clarified that rather than every trimer being bound by two Fabs, there was a distribution of binding stoichiometries, demonstrating the power of single particle methods and the potential ambiguity of ensemble methods. We have attributed substoichiometric binding of Fabs to Env to the heterogeneous glycans on the surface of the trimer restricting access to nAb-binding sites to various extents (2, 11, 20).

Here, we used NS-EM to examine binding kinetics and the phenomenon of substoichiometric binding with a more systematic approach. We used two CD4bs bnAbs VRC01 and 3BNC117, both related to PGV04, and BG505 SOSIP.664 trimers in an attempt to gain a better understanding of the bnAb–Env interaction at a single particle level. Based on the published kinetic parameter differences (19), as well as insights into the nuances of the Env-binding mechanisms for these two bnAbs (*i.e.* the extent to which they engage Env glycans) (15), we hypothesized that the trimer occupancy distributions for the bnAbs may be a function of both the bnAb and time, and are not entirely stochastic or simply an artifact of inconsistent glycosylation patterns within Env protein preps. Moreover, through the single-particle “visual” kinetic analysis of these VRC01-class Fabs binding to trimers with epitope-modifying mutations, we also sought to determine the value of observing protein-protein interactions on longer-range time scales.

## Results

### Time-course negative-stain EM of VRC01-class bnAb–Env interactions

We used VRC01 and 3BNC117 Fabs and the BG505 SOSIP.664 trimer as the model system for evaluating the NS-EM method for visualizing protein–protein interactions. We chose these two Fabs because, in addition to being clinical candidates, they bind the same CD4bs region of the Env, using conserved residues, yet they display divergent biophysical characteristics. In most studies 3BNC117 shows higher affinity and stoichiometry than VRC01 (2, 4–10, 15, 19). These antibodies, like most bnAbs, are also characterized by relatively modest on-rates and negligible off-rates. Indeed, our BLI results obtained using VRC01 and 3BNC117 IgG's immobilized onto anti-hIgG Fc Capture (AHC) sensors, with the BG505 SOSIP.664 trimer as the analyte, show that both bound the trimer with subnanomolar affinity, with the 3BNC117 *K_D_* approximately half that of VRC01 (Table 2).

**Table 2:** **Biolayer interferometry-derived kinetic parameters for VRC01 and 3BNC117 IgG binding to BG505 SOSIP.664**

IgG	*K_D_*	*K_D_* error	*K*_on_	*K*_on_ error	*K*_off_	*K*_off_ error
**3BNC117**	1.91 × 10^−10^	5.27 × 10^−12^	2.15 × 10^4^	39.5	4.11 × 10^−6^	1.13 × 10^−7^
**VRC01**	4.60 × 10^−10^	6.60 × 10^−12^	1.99 × 10^4^	40.9	9.14 × 10^−6^	1.30 × 10^−7^

**Figure 1.**
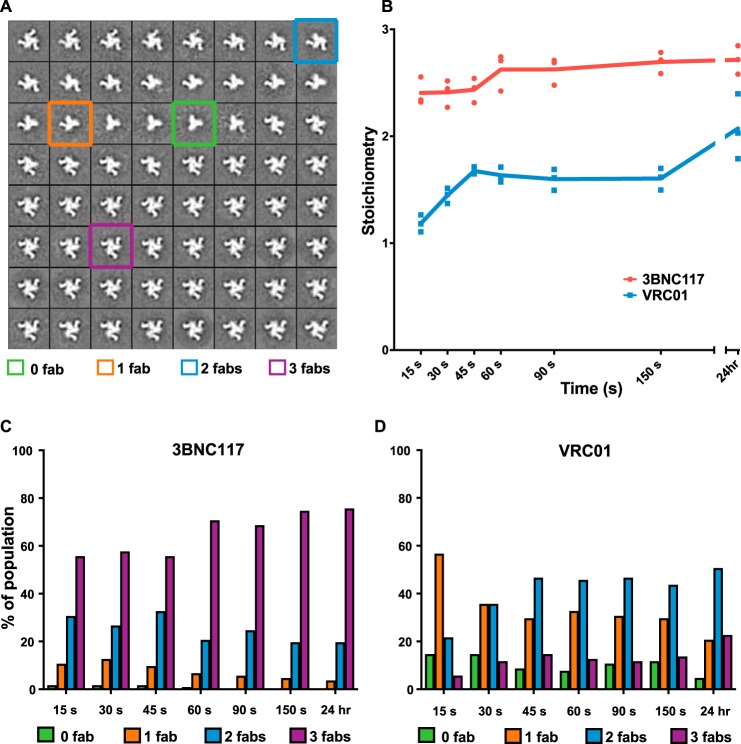
*A,* sample 2D class averages used to determine average stoichiometry for each time point, with various occupancy level examples highlighted. *B,* stoichiometry of VRC01 and 3BNC117 binding to BG505 SOSIP.664 as measured using negative-stain EM. All time points were generated using 3 grid preps (technical repeats); *dots* indicate the individual technical repeat stoichiometry results that were used to calculate the means reported in the text. A third of the VRC01 data shown for 15 to 150 s was also generated with independent BG505 SOSIP.664/Fab expression/purifications thus the scatter in the data also reflects variability of biological replicates (*i.e.* one data point within each time point was generated with proteins prepared on different days). *C,* distribution of BG505 SOSIP.664 trimer-3BNC117 stoichiometry across time points ranging from 15 s to 24 h. *D,* distribution of BG505 SOSIP.664 trimer-VRC01 stoichiometry across time points ranging from 15 s to 24 h (% values are the average of 3 technical repeats, see supplemental Table S1 for variability estimates).

Although the aforementioned cryo-EM results were informative (11) they only represented a single observation under equilibrium conditions and did not provide insight into the affinity or whether the Fabs might bind the trimer in a cooperative manner. We therefore imaged the trimer with 6-fold excess of Fab (∼600 nm Fab to ∼90 nm trimer) at time intervals ranging from 15 s to 24 h post-mixing to generate a visual binding profile. Particles were picked from raw images, stacks created, and reference-free 2D classification was undertaken (see “Experimental procedures” for details). Because the data were dominated by primarily top views of the complex it was easy to distinguish among the various subpopulations of particles. Thus, at this stage, the occupancy of Fabs per trimer was evaluated visually in the 2D class averages, with the possible classes being trimers with 0, 1, 2, or 3 Fabs bound (Fig. 1*A*). A subset of the data were generated using trimer protein expressed and purified on different days, and these suggested there was no additional impact of the protein prep on the variability in occupancy level and distribution on top of the inherent variance associated with the technical repeats (see Fig. 1*B*).

Fig. 1*B* shows a time course of Env binding to either VRC01 or 3BNC117, illustrating the differences in stoichiometries at all time points examined, with an average steady-state stoichiometry of 1.92 and 2.70, respectively, which is close to the previously reported SPR-derived S_m_ values of 1.6 and 3.0, respectively (15). Here again, EM 2D classification deconvoluted the stoichiometry of binding into an ensemble of classes. The average number of particles examined per time point was ∼65,000 for 3BNC117 and ∼67,000 for VRC01. Fig. 1*C* and supplemental Table S1 show how trimers of varying 3BNC117 Fab occupancy varied as a function of time, with roughly half of the trimers (56%) fully occupied by the 15-s time point and 73% after 60 s. In the VRC01 case, on the other hand, the fully occupied trimers represented only 6% of the particles at 15 s and never surpassed 23%, even after 24 h incubation (Fig. 1*D*, supplemental Table S1). Indeed, the predominant VRC01 class was the 2-Fab-bound trimer at ∼47% after 45 s, and stayed around the 50% level even out to 24 h. This result led us to question whether the frequently used 6-fold excess of Fab to trimer is sufficient to achieve maximum saturation of the trimer with either Fab (11, 13, 15).

**Figure 2.**
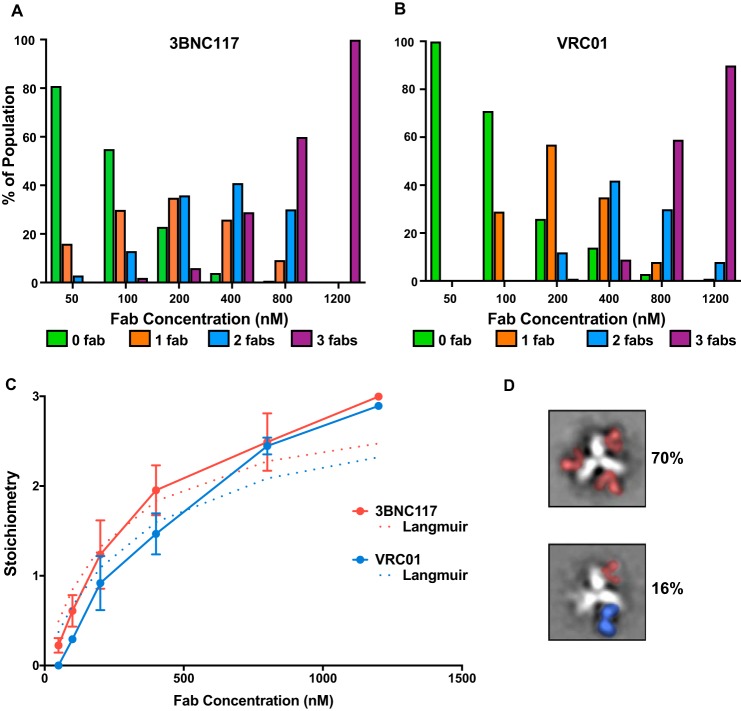
*A* and *B*, BG505 SOSIP.664 trimer-Fab occupancy distribution as measured using negative-stain EM at increasing Fab concentrations for (*A*) 3BNC117 and (*B*) VRC01. *C,* stoichiometry as a function of Fab concentration. Data are presented as the mean of 3 technical repeats ± S.D., except for 800 nm 3BNC117 where 6 technical repeats were performed due to larger than expected variability. Langmuir isotherm least squares fit (*dashed lines*) was applied to the stoichiometry values for 3BNC117 and VRC01 data (R^2^ = 0.98 and 0.99, respectively). *D,* representative sCD4-bound class averages resulting when 600 nm VRC01-preincubated BG505 SOSIP.664 trimer was allowed to interact with 600 nm sCD4 overnight, showing the predominant (70%) class average was the CD4-bound open conformation (*top*; sCD4 and V1/V2 loops in *red*), with 16% of the population mixed (*i.e.* some combination of a single VRC01 Fab (*blue*) bound and sCD4).

We therefore imaged the trimer (∼90 nm) after a 1-h incubation in the presence of increasing amounts of Fabs. 3BNC117 samples again exhibited higher occupancy relative to VRC01, but this phenotype diminished at Fab concentrations >800 nm (Fig. 2). At the highest Fab concentration of 1200 nm there is little difference in the global stoichiometry with both samples showing predominantly the 3-Fab-bound trimer. Notably, however, VRC01 still does not fully saturate the BG505 SOSIP.664 trimer, with 10% of the particle population still in the incompletely occupied state (primarily 2-Fab-bound trimer; Fig. 2*B*). The increased occupancy of VRC01 observed at concentrations >800 nm may indicate the presence of a lower affinity epitope on some trimers due to variable glycosylation proximal to the CD4bs. The stoichiometry-based binding curves (Fig. 2*C*) also do not exclude the possibility of a cooperative binding mechanism, as they do not follow the classic exponential shape of the 1:1 binding isotherm. Indeed, the Langmuir isotherm underestimated the maximum stoichiometry for both Fabs by ∼20%, likely because it did not account for the inflection point that is apparent for 3BNC117, in particular (Fig. 2*C*).

To gain insight into whether HIV trimers that are not fully occupied by VRC01 are still capable of engaging the CD4 receptor, we incubated the BG505 SOSIP.664 trimer (at 90 nm) with 600 nm VRC01 for 1 h, followed by overnight incubation with 600 nm soluble CD4 (sCD4). Interestingly, in 70% of the 135,000 particles counted, the predominant class of particle was the sCD4-bound open trimer, with mixed sCD4-VRC01 classes making up ∼16% and 1-, 2-, or 3-Fab-bound trimers making up the remainder (Fig. 2*D*). We then performed the same experiment but using 3BNC117. In this case, we did not observe any sCD4 bound to BG505, with ∼30,000 particles examined. These data imply that VRC01 dissociates from the trimer, whereas 3BNC117 binds almost irreversibly, despite both displaying similarly low off-rates during the 1-h measurement in BLI. Hence, 3BNC117 may be the better candidate for therapeutic vaccination.

### Effect of Env mutations around the CD4bs on stoichiometry time course

**Figure 3.**
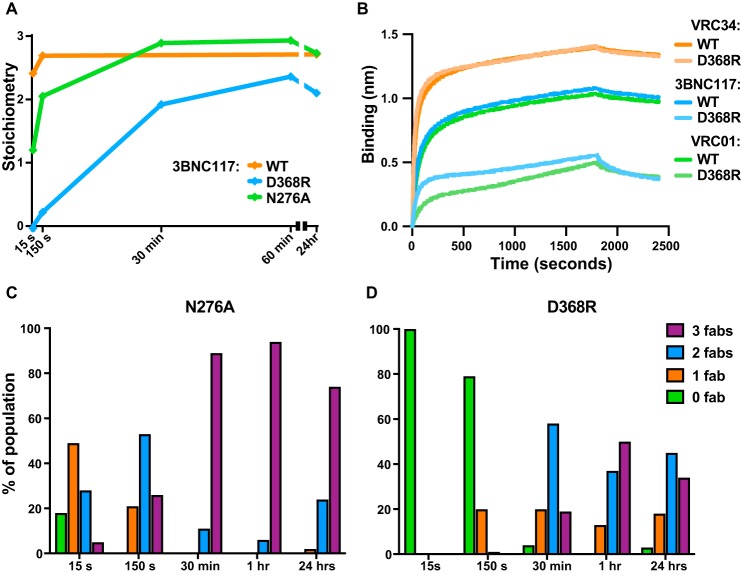
*A,* stoichiometry of BG505 SOSIP.664 trimer-3BNC117 Fab binding at multiple time points as observed with negative-stain EM. WT SOSIP was performed in triplicate (*n* = 3), and a single grid (*n* = 1) was examined for the N276A and D368R mutants (15,000–69,000 particles per time point). *B,* Octet raw data traces comparing binding of 3BNC117 and VRC01 to WT or D368R BG505 SOSIP.664 trimers. *C* and *D,* trimer occupancy of 3BNC117 Fab distribution at multiple time points for the N276A and D368R mutants, respectively.

Because of the high-level of interest in CD4bs bnAbs a number of mutations have been identified that alter binding. To test the sensitivity of the negative-stain EM visual kinetic analysis to mutations in and around the CD4bs, we generated two BG505 SOSIP.664 mutants. The first, D368R, removes the essential aspartic acid in the heart of the CD4bs that the HIV uses to bind the CD4 receptor, and one which VRC01-class antibodies have evolved to engage as part of their CD4 mimicry mechanism via the HC Arg-71 (21, 22). Previous ELISA results have suggested that this mutation abrogates VRC01-class bnAb-Env binding (15). In our EM analysis, however, 3BNC117 Fab is still capable of binding the D368R trimer in solution, albeit with apparently reduced affinity and stoichiometry, with no Fabs binding the trimer at 15 s (out of a 16,600-particle sample), and a small population bound at the 150-s time point that was severely reduced relative to the average stoichiometry of the WT (D368R, 0.22 *versus,* WT, 2.69; Fig. 3*A*). 3BNC117 is ultimately able to bind the modified trimer with an average steady-state stoichiometry reduced by 20% relative to WT, with the associated trimer occupancy distribution in Fig. 3*D* suggesting that the steady-state dynamic equilibrium is also shifted toward lower-occupancy relative to WT (Fig. 1*C*), highlighting the pivotal role that Asp-368 plays in the VRC01-class bnAb binding mechanism. Thus, our EM data suggest that although the D368R mutation substantially decreases the affinity it does not completely eliminate binding. Moreover, using 3BNC117 and VRC01 IgGs as the ligands, BLI did confirm a marked decrease in binding to the D368R BG505 SOSIP.664 trimer in the 30-min association step, whereas a control bnAb, VRC34, that binds in the fusion peptide region outside the CD4bs, bound the D368R mutant with similar affinity to the WT SOSIP (Fig. 3*B*).

The next mutation that we tested was N276A that results in the deletion of a glycan that is peripheral to the CD4bs and a key impediment to accessing the conserved receptor-binding site epitope (20). Using SPR, Behrens *et al.* (19) noted that removal of this glycan increases the *k*_on_ but also increases the *k*_off_ for both 3BNC117 and VRC01, reducing their overall affinities. Thus, whereas the Asn-276 glycan is an entropic barrier to initial binding, the antibody makes specific interaction with the glycan to achieve high affinity. We recently characterized this type of interaction as a “glycan clasp” in another HIV antibody (16). Our NS-EM results suggest removal of the Asn-276 glycan slows the rate at which 3BNC117 is able to reach its steady-state occupancy level, perhaps due to the increased *k*_off_ when the glycan is deleted (Fig. 3, *A–C*). Furthermore, steady-state occupancy is reached between 150 s and 30 min, compared with 45 s to 60 s for the WT (Fig. 1*C*). Examining the occupancy distribution shifts, it is also evident that the fully occupied trimer is underrepresented in the N276A sample relative to the WT in the earlier time points (*i.e.* 15 and 150 s). We hypothesized that the increased *k*_off_ of 3BNC117 when Asn-276 was deleted may make the 3BNC117-bound BG505 trimer susceptible to CD4 binding. Surprisingly, the mutated trimer bound to 3BNC117 was still resistant to CD4 binding (data not shown).

### Multivariate statistical analysis/multireference alignments (MSA/MRA) versus RELION 1.4 comparison

**Figure 4.**
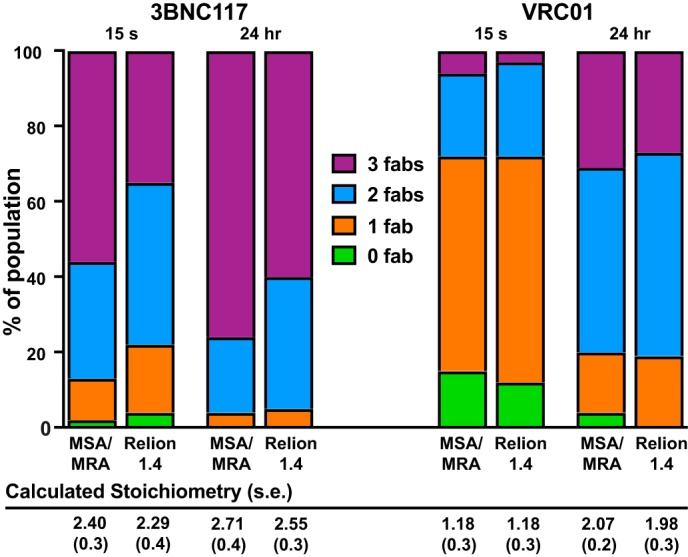
**BG505 SOSIP.664 trimer–Fab occupancy distribution comparison between MSA/MRA and RELION 1.4 for 3BNC117 and VRC01 at 15 s (*left*) and 24 h (*right*) (*n* = 3 for each).**

To rule out the possibility of 2D classification bias, a subset of the time points for both VRC01 and 3BNC117 was subjected to 2D classification using two different algorithms, MSA/MRA and RELION 1.4 (23, 24). The classification results were similar for both the 15-s and 24-h time points (Fig. 4). The MSA/MRA *versus* RELION 1.4 15-s and 24-h average occupancies for VRC01 and 3BNC117 were within experimental error of each other (∼0.2–0.4). Moreover, the distribution of the subpopulations of trimers followed a similar trend, although the RELION processing package appeared to favor classification of a larger number of lower-occupancy trimers in the case of 3BNC117. The overall trimer occupancy varied by ≤5% between the two methods for this Fab and ≤3% for VRC01. The seemingly increased tendency for RELION to populate an increased number of under-saturated trimers was likely due to the algorithm's finer 2D alignment ability to resolve 1-Fab and 2-Fab trimers that may have been classified into the 3-Fab trimer class using the “coarser” MSA/MRA alignment methodology. Indeed, it is known that over-fitting can be an issue for low-resolution negative-stain data, thus the RELION program provides an option to limit the resolution in the E-step (*i.e.* the alignment). We found that setting a higher threshold (*e.g.* 20 Å) than the default (no limit) results in the occupancy and stoichiometry data generated by RELION 1.4 converging into those values obtained with MSA/MRA. There are multiple other factors (*e.g.* number of classes requested from either alignment algorithm) that can shift trimer occupancy distribution balance *slightly* either way between the two approaches, but it is important to note that the comparison in Fig. 4 suggests that the trends in stoichiometry over time for each Fab, as well as the shifts in trimer occupancy distributions are consistent between 2D alignment algorithms. Indeed, in the case of VRC01, both the MSA/MRA *versus* RELION 1.4 average stoichiometry and the trimer occupancy distributions were essentially identical for both the 15-s and the 24-h time points.

### Automated trimer sorting model validation

**Figure 5.**
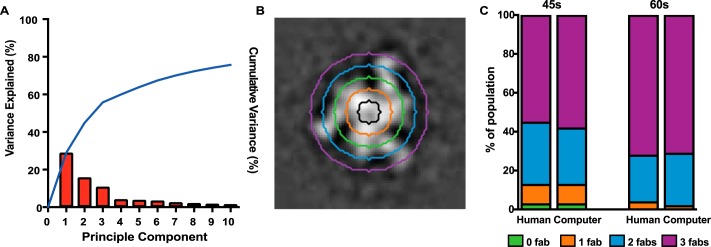
**Automated prediction using concentric Fourier sampling on class averages.** *A,* Pareto diagram showing variance explained using principle component analysis of synthetically trained classifier. *B,* example class average showing concentric sampling pattern (original *grayscale* image shown). *C,* predicted distributions of Fab occupancy *versus* actual distribution for two example datasets. In both cases the automated classifier performs with greater than 92% accuracy in predicting occupancy.

Although the complexes above were initially analyzed via visual inspection, we endeavored to create a more objective and faster method for determining stoichiometry. This method is based around a new approach to particle or object information representation referred to as Bag of Frequencies, which has been applied to tilt-series images of nodules in the lungs with some success (25). Our specific approach is simpler as we are working in 2D space as opposed to 3D space. By extracting concentric rings originating from the center of the class average images (Fig. 5*B*), treating them as periodic signals, and extracting binned information regarding signals present in the Fourier transform, we are able to obtain a rotationally invariant representation of the image data that, due to the nature of the HIV trimers used and binding sites surveyed, allows us to sort trimers with high success using classical machine learning methods trained on synthetic data models. Automated models were tested on two empirical datasets (3BNC117 45- and 60-s time points, see Fig. 1*C*) to test efficacy in real-world situations. The results are shown in Fig. 5*C*. The 45-s time point dataset contained 154 class averages representing all occupancy levels, and all occupancy levels were conserved in automated prediction. The model's predictions in this case were 94.16% accurate, relative to expert human assignment, and conserved the distribution of occupancy. The 60-s time point dataset contained 131 class averages at single, double, and triple occupancy levels, which were conserved in automated prediction. The model's predictions were 93.18% accurate, relative to expert human assignment, and conserved the distribution of occupancy. Thus, these methods can potentially remove bias and increase throughput of data analysis.

## Discussion

The goal of this study was to explore the potential of a NS-EM technique to add a more nuanced understanding of the dynamics of bnAb–Env interactions and discriminate between members of highly related classes of bnAbs. The single particle method is based on imaging negatively stained EM grids at multiple time intervals to capture snapshots of intermediate interaction states, and it enables a systematic stratification of the observed particle species into distinct 2D subclasses. In a similar time-resolved NS-EM approach, Mulder *et al.* (26) monitored the population flux of 14 30S ribosomal subunit assembly intermediates over the course of 120 min, resulting in a more comprehensive understanding of the ribosome assembly mechanism. In a similar application, the Frank lab was successful in resolving distinct pre-equilibrium ribosome 3D structural conformations on the millisecond time scale (27). For our purposes negative-stain EM was actually advantageous because the particles adhered to the carbon substrate in one predominate fashion and this preferred orientation enabled robust quantification of Fab binding.

Using CD4bs bnAbs and the Env trimer as our model system, we performed the visual analysis of binding interactions at the 2D class-average level to probe differential levels of bnAb saturation of the Env trimer. Because our model system did not require cryo-EM or 3D reconstruction, the technique also lent itself to the potential automation of occupancy classification. Furthermore, by examining the binding of these Fabs to select Env mutants, we demonstrated the potential of this technique to detect interaction states that may be missed entirely by ensemble methods such as ELISAs.

**Figure 6.**
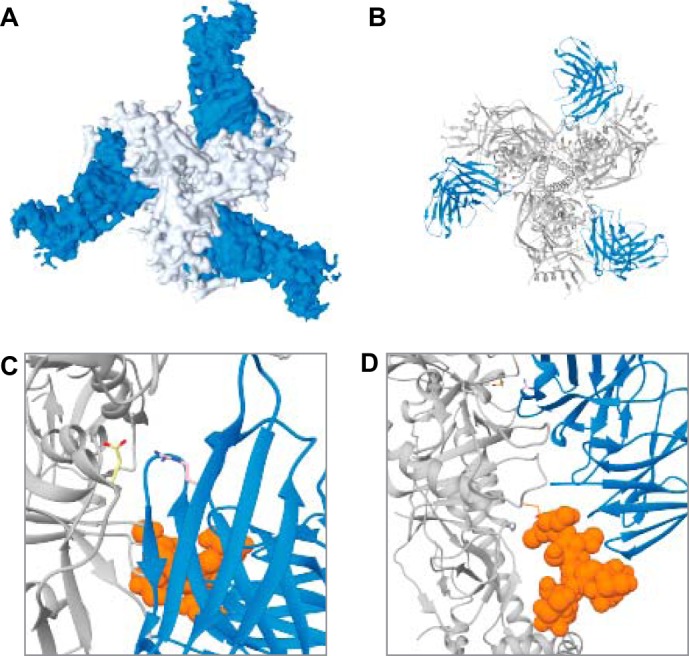
*A* and *B,* top view of BG505 SOSIP.664 trimer bound by three 3BNC117 Fabs bound in surface and ribbon representation, respectively. *C,* top view of interaction between Asp-368 in gp120 and Arg-71 in 3BNC117. *D,* side view of Asn-276gp120 glycan interaction with 3BNC117 light chain.

The two bnAbs used here derive from the same germline precursor and implement a nearly identical binding mode to Env (22, 28). Fig. 6, *A* and *B,* shows a representative 3D reconstruction of the BG505 SOSIP.664 trimer bound by three 3BNC117 Fabs. Both the CD4bs Fabs' variable heavy chains mimic the Arg-59_CD4_–Asp-368_gp120_ interaction via their HC Arg-71 residue (Fig. 6*C*) (28, 29). Despite their very similar gp120 interactions, however, VRC01 and 3BNC117 have been shown to exhibit differences in their interaction kinetics with the viral spike, possibly due to the differences in how they engage and/or avoid the glycans that surround the CD4bs (15, 19, 29, 30). Although both have low nanomolar affinities for their target (see Table 2), 3BNC117 has been shown to have higher *k*_on_ and binds with higher occupancy based on SPR data (19). Still, techniques such as SPR, BLI, and ITC produce singular quantitative parameters such as a stoichiometry value, S_m_, that are the steady-state average of an ensemble of the population examined and give no indication of the distribution of occupancy states over the course of time. Such information may be of value in cases where significant impediments to binding exist (*e.g.* multiple glycans) as these obstructions may extend the opportune time window to observe intermediate states to the second, or even minute-based time scale.

Our NS-EM experiments confirmed that VRC01 and 3BNC117 bind Env differently. The data also suggest when steady-state is reached in our solution-based system and provided insight into trimer occupancy shifts over time. Although the exact timing of the steady-state binding stoichiometry of either of the Fabs to Env may not be paramount (as it may be specific to a particular subtype of HIV Env), we highlight this visual kinetics difference between the two Fabs in the context of what is already known about their interaction with Env. Namely, we show that 3BNC117 is better at negotiating Env glycosylation to bind the trimer with both higher occupancy, faster on-rates, and lower off-rates, and the same Asn-276 glycan that 3BNC117 uses to enhance its binding of the trimer (Fig. 6*D*) may present a greater impediment to VRC01 (15). Here, when the Fab concentration was increased to a micromolar level, both Fabs reach similar levels of average steady-state stoichiometry. These data suggest a low-affinity secondary binding event that may occur when both Fabs approach micromolar concentrations, thus partly masking 3BNC117's superior ability to navigate the extensive glycan shield (Fig. 2*C*) (19). Still, VRC01 does not fully saturate the trimer (Fig. 2*B*), and this may suggest that the glycan impediment to VRC01 cannot be entirely overcome through artificially increased avidity, and a subset of the particles may always remain under-occupied. The ability to fully saturate the HIV trimer may in part explain why 3BNC117 is a more broad and potent HIV antibody (31), which, in addition to its glycan interaction advantages, may also be explained by an increased number of putative contact residues in the heavy chain framework region around the CD4bs (15, 16, 28, 32).

Our sCD4 competition experiment was consistent with these observations, because the BG505 SOSIP.664 trimer was apparently unable to engage this soluble version of the CD4 receptor when preincubated with 3BNC117, whereas preincubation with VRC01 did not prevent sCD4 binding (Fig. 2*D*). Although somewhat surprising, the VRC01 result was not entirely unexpected based on the aforementioned structural details of the respective bnAb–gp120 interactions. Additionally, clinical data demonstrated that a multiple VRC01 IgG dosing regimen was not effective in preventing viral rebound after treatment interruption, and it has been suggested that chronic infectees may frequently exhibit increased levels of archived VRC01-resistant virus relative to 3BNC117 (33). Conversely, in two separate trials, 3BNC117 was efficacious in preventing both viral rebound and significantly reduced HIV load for about 1 month with a single infusion (7, 8).

The results in Fig. 2, *A* and *B,* and the concentration curve in Fig. 2*C* suggest that, variably glycosylated trimers are likely not the sole reason for observing a distribution of occupancy states because 3BNC117, in contrast to VRC01, was capable of 100% occupancy at micromolar concentrations. Rather, in this proof-of-concept study, the differences in the distribution appear to be at least in part influenced by the nuances of the binding mechanisms for these two bnAbs. Indeed, previously published data shows that the removal of the Asn-276 glycan doubles the VRC01 Fab's *k*_on_ and increases the average S_m_ from 1.6 to 2.5, whereas also significantly increasing the *k*_off_ thereby decreasing *K_D_*. 3BNC117 showed a similar trend, albeit with a much smaller effect on the *k*_off_ and *K_D_*, perhaps because of the additional gp120 contact residues within its CD4bs paratope (19, 28). Previous ELISA results also suggested that knocking out this key glycosylation site reduced Env binding to 3BNC117 IgG (15).

In our NS-EM experiments, 3BNC117 struggled to remain bound to the N276A glycan knock-out. These results are in agreement with the analogous comparison of Behrens *et al.* (19) of the same Fab and trimer via SPR, where 3BNC117 ultimately does reach a steady-state average N276A trimer occupancy similar to that of the WT trimer (Fig. 3*A*). It is also important to highlight a key distinction between the qualitative single-particle data obtained here and Behrens *et al.* (19) N276A mutant *versus* WT BG505 SOSIP.664 SPR data, as our EM and their SPR results may seem contradictory at face value. Indeed, the steeper N276A SPR binding curve associated with faster on-rates may be a reflection of the enhanced instantaneous ability of the Fab in solution to bind the immobilized trimers, with each binding event occurring more rapidly relative to WT due to decreased CD4bs obstruction with the glycan. The absence of this same glycan, however, is also responsible for the increase in the off-rate, with the net effect of decreasing the *K_D_*. Our single-particle data, on the other hand, provides snapshots of intermediate occupancy states of the free trimer in solution that are a visual representation of a balancing act between increased on- and off-rates and may thus be reflective of the net decreased affinity at each time point prior to reaching steady-state.

Interestingly, we were still unable to observe any sCD4-bound N276A BG505 SOSIP.664 trimers when preincubated with 3BNC117. This result suggests that the SPR-observed increase in *k*_off_, and the concomitant affinity decrease due to the glycan removal (19), are not sufficient to substantially affect 3BNC117's potency/function. Indeed it appears that the Fab's ability to saturate the modified trimer given sufficient time (Fig. 3*A*) is the predominant factor in determining its capacity to block CD4 binding to the trimer.

The advantage of our visual single-particle technique is that one can potentially observe protein–protein interaction states that may otherwise be hidden within the averaged signal output by molecular ensemble-based kinetics methods. We demonstrate this in Fig. 3, *A, B*, and *D,* via the D368R BG505 SOSIP.664 mutant, because previous results have indicated that 3BNC117 IgG may be unable to bind Env with this aspartic acid removed (15). Combined with our BLI results, the single-particle observations do indeed suggest that antibody binding to this modified trimer is compromised, with the stoichiometry time course as well as the trimer occupancy distribution, offering a more nuanced view of the modified protein–protein interaction. Because 3BNC117 is ultimately able to bind the modified trimer, albeit with reduced stoichiometry and the associated occupancy subpopulation shifts (Fig. 3*D*), another key inference here is that kinetics measurement methods that do not allow for sufficient interaction time for the protein–ligand interaction may miss the longer range time scale interactions that may be present in systems where heavily glycosylated and certain intrinsically disordered protein-binding partners are involved (34). Indeed, with the association step extended to a lengthy 30 min, our BLI result was also suggestive of a modified binding mechanism to the D368R mutant in the case of 3BNC117 and VRC01, with a discernable inflection point suggestive of a secondary binding event around 500 s, which is temporally similar to the emergence of double and triple occupancy trimers in our EM dataset (Fig. 3, *B–D*).

To further facilitate a higher throughput application of this visual kinetics method, we tested an automated classification approach for predicting trimer occupancy with relative success. Our initial tests of automated classification based on Support Vector Machines and Concentric Fourier Sampling performed with ∼94% accuracy relative to human benchmarks, paired with conservation of occupancy distribution (Fig. 5*C*). We note that future improvements in accuracy may be achieved by modifying training datasets to contain more realistic representations of the empirical data, either through incorporation of actual class averages, or modified synthetic data. We plan to expand testing into more diverse datasets to test efficacy on varying proportions of occupancy, as well as different total sample sizes. Once further validation is complete, we hope that this will enable further automation of the method.

In summary, we describe here a unique approach for interrogating protein–protein interactions on a single-particle level. By visually assessing the stoichiometry of CD4bs Fab binding to the HIV Env trimer over time, we demonstrate that, whereas certain steady-state ensemble kinetic method-derived constants are indeed observed on the single-particle level, detailed information about the distribution of protein–protein interaction states may be missed by bulk solution-based kinetics measurements. This relatively simple method may prove particularly useful in systems where significant barriers to binding and complex dynamic equilibria exist, thus extending the opportune window for observing protein–protein interaction states that may go unnoticed with traditional kinetics measurement methods.

## Experimental procedures

### Recombinant protein expression and formation of the Fab–Env complexes

3BNC117, VRC01 Fabs, and BG505 SOSIP.664 (±D368R/N276A) trimers were expressed in 293F cells and purified using a previously described procedure (16). Briefly, the trimers were affinity-purified using 2G12 antibody resin, buffer exchanged into 20 mm Tris, 0.5 m NaCl, pH 8.0 (TBS), followed by removal of aggregates, monomers, and dimers via size exclusion chromatography (SEC). If necessary, the SEC-purified trimers were further concentrated using Amicon Ultra-4 Centrifugal filter Devices (100,000 MWCO) for biophysical analyses. The Fabs were affinity-purified with Lambda Select resin (GE Healthcare) followed by cation exchange chromatography to enrich for the correct HC/LC dimers, which were then further purified/buffer exchanged into TBS using SEC. Trimer–Fab complexes were formed using a range of molar ratios. For equilibrium experiments, a ∼6-m excess of Fab was added to the trimer (∼90 nm) under ambient conditions and applied to carbon-coated copper grids at time points ranging from 15 s to 24 h. To generate concentration curves, samples with 50–1200 nm Fab were prepared while keeping the trimer concentration constant; these samples were incubated for 1-h under ambient conditions. For the sCD4 experiment, 6 m excess Fab was incubated with the trimer (90 nm) for 1 h, followed by an overnight incubation with 6 m excess sCD4.

### Electron microscopy

At time points ranging from 15 s to 24 h, the Fab–trimer mixture was applied to glow-discharged, carbon-coated 400-mesh copper grids as follows. The Fab–trimer mixture was diluted with TBS to a trimer and Fab concentration of ∼91 and 600 nm, respectively. A time point was defined as the time the sample was deposited on the grid after incubation of the trimer–bnAb. For the concentration-based experiments, the trimer concentration was kept constant at 91 nm, whereas the Fab concentration was increased from 50 to 1200 nm, with each sample incubated for 1 h. Staining was performed by applying 3 μl of 2% (w/v) uranyl formate stain and blotting off immediately, followed by application of another 3 μl of stain for 45–60 s, followed by blotting. Stained grids were allowed to air-dry and stored under ambient conditions until ready for imaging. Images were collected via Leginon software (35, 36) using FEI Talos, Tecnai F20, or Tecnai T12 electron microscopes operated at, respectively, 200 kV ×73,000, 200 kV ×55,000, and 120 kV ×52,000 magnification. In all cases, the electron dose was 25 *e*^−^/A2. Particles were picked from the raw images using DoG Picker (37) and placed into stacks using Appion software (38). Finally, 2D reference-free alignment was performed using iterative MSA/MRA)(23). Distribution of 0, 1-, 2-, or 3-Fab bound trimers were visually determined by counting the number of total particles (in the top or bottom view) belonging to each category on the 2D class average level for each experiment. RELION 1.4 was used to cross-corroborate the 2D classification results for a subset of the data. Briefly, the particle stacks for the 15-s and 24-h time points (*n* = 3 for each time point) were converted from IMAGIC to RELION-formatted MRC stacks and subjected to RELION 2D classification (24).

### Octet experiments

The Octet Red biolayer interferometry platform was used for the binding experiment shown in Fig. 3*B* and for generating the kinetic parameters in Table 2. IgGs were immobilized on activated anti-human IgG Fc sensors (AHC: ForteBio) and BG505 SOSIP.664 trimers were used as the analyte. The AHC biosensors were immersed in PBS, 0.1% BSA, 0.002% Tween 20 (pH 7.4) containing 10 μg/ml of IgG (VRC01 or 3BNC117) until a signal threshold of 1 nm was reached for at least 1 sensor. To generate the baseline kinetic parameters shown in Table 2, the SOSIP concentrations ranged from 500 to 7.8 nm and all steps of the assay occurred with the plate vibration set to 800 rpm. The association and dissociation step duration was set to 800 and 3600 s, respectively. For the D368R SOSIP experiment in Fig. 3*B*, assay set-up was the same except the association and dissociation times were set to 1800 and 600 s, respectively, and only a single concentration of analyte (250 nm) was used.

### Generation of synthetic data for automated sorting

We prepared synthetic representations of class average data by obtaining the 3D volume of the ZM197 SOSIP.664 HIV-1 trimer with three VRC01 antibodies bound at the CD4-binding site (PDBE EMD-3059). This volume was then progressively modified in UCSF Chimera's (39) segmentation tool to represent 0, 1, and 2 Fab-bound trimer states. The volumes were then rotated at 10 degree intervals from 0 to 120 degrees and projected in 2D space from the top view. EMAN (40) was then used to apply small random translational shifts (+ −2px *x*/*y*) and a large radius Gaussian filter to simulate noise. This resulted in 960 synthetic class averages with an even distribution of antibody occupancy representation.

### Automated trimer sorting based on occupancy

Automated sorting was performed in MATLAB R2016b using the Image Processing and Statistics and Machine Learning toolboxes. Input particles are assumed to be properly centered during the picking and class average creation steps. We use a sampling method similar to the initial processing steps of the Bag-of-Frequencies methodology successfully implemented for classifying images from lung CT scans (25). This concentric Fourier sampling is performed by sampling each image in a circular pattern originating from the center of the image in five pixel intervals from five to 25 pixels (Fig. 5*B*). The fast Fourier transforms of the linearized concentric samples are then calculated and transformed into single-sided amplitude spectra. The spectra are concatenated together into an array and used as input for Support Vector Machine (SVM) classification. Validation of the SVM classifier was performed by 10-fold cross-validation, and the data passed into the SVM classifier was analyzed using principle component analysis. The model scored >99% accuracy in 10-fold cross-validation. Principal component analysis of the data fed into the classifier shows that whereas there are three Fourier bins that explain >60% of the total variance in the data, there is meaningful variance spread throughout the dimensions of the data, and as such the entirety of the data are used in classification for testing purposes (Fig. 5*A*). Testing performance of the classifier on real world datasets was performed by using the entire synthetic dataset to train the predictive SVM model, and passing through the concentric Fourier sampling information for test class averages.

## Author contributions

A. B. W. and B. N. conceived and designed the experiments. B. N. performed the experiments. C. A. B. performed the automated trimer classification. B. N., A. B. W., and C. A. B. wrote the paper.

## Supplementary Material

Supplemental Data

## Footnotes

2 The abbreviations used are:

ITC: isothermal calorimetry
BLI: biolayer interferometry
S_m_: stoichiometry
NS-EM: negative-stain electron microscopy
Env: HIV envelope glycoprotein
bnAb: broadly neutralizing antibody
cryo-EM: cryo-electron microscopy
CD4bs: CD4-binding site
FAB: fragment antigen binding
AHC: anti-hIgG Fc capture
sCD4: soluble CD4
MSA: multivariate statistical analysis
MRA: multireference alignments
SVM: support vector machine.
