# Supplementary material for: Time-course, negative-stain electron microscopy–based analysis for investigating protein–protein interactions at the single-molecule level
Source: J Biol Chem. 2017 Sep 29;292(47):19400–10. doi: 10.1074/jbc.M117.808352 (PMC5702678; doi:10.1074/jbc.M117.808352)
Supplement: Supplemental Data [file supp_292_47_19400__index.html]

Time-course, negative−stain electron microscopy−based analysis for investigating protein−protein interactions at the single−molecule level — Time-course, negative-stain electron microscopy–based analysis for investigating protein–protein interactions at the single-molecule level — Single-particle kinetics using NS-EM — Supplemental Data 

# Time-course, negative-stain electron microscopy–based analysis for investigating protein–protein interactions at the single-molecule level

## Supplemental Data

- Supplemental Data (.pdf, 93 KB) - Supplemental Data
