# Supplementary material for: Time-course, negative-stain electron microscopy–based analysis for investigating protein–protein interactions at the single-molecule level
Source: J Biol Chem. 2017 Sep 29;292(47):19400–10. doi: 10.1074/jbc.M117.808352 (PMC5702678; doi:10.1074/jbc.M117.808352)
Supplement: Supplemental Data [file 10.1074_M117.808352_jbc.M117.808352-1.pdf]

Time-Course Negative Stain Electron Microscopy-Based Analysis Enables Unique Insight into HIV Envelope Trimer-Ab Interaction States

**Bartek Nogal, Charles A. Bowman, and Andrew B. Ward**

**Table S1. BG505 SOSIP.664 trimer occupancy distribution and associated variability using VRC01 and 3BNC117 Fabs incubated with the trimer over increasing durations.**

**Table S1. BG505 SOSIP.664 trimer occupancy distribution and associated variability using VRC01 and 3BNC117 Fabs incubated with the trimer over increasing durations.**

| | VRC01 | | | | | | 3BNC117 | | | | | |
|---|---|---|---|---|---|---|---|---|---|---|---|---|
| | | Fabs bound to trimer (% ± S.E.) | | | | | | Fabs bound to trimer (% ± S.E.) | | | | |
| **Time** | **Total # of Particles** | **0** | **1** | **2** | **3** | **S** | **Total # of Particles** | **0** | **1** | **2** | **3** | **S** |
| **15s** | 40352 | 15.4 ± 1.18% | 56.6 ± 3.78% | 22.1 ± 4.76% | 5.83 ± 1.41% | 1.18 | 50696 | 1.90 ± 0.460% | 11.4 ± 2.11% | 30.9 ± 3.87% | 55.8 ± 5.61% | 2.4 |
| **30s** | 31012 | 15.3 ± 2.23% | 36.4 ± 1.64% | 36.3 ± 2.04% | 11.9 ± 2.47% | 1.45 | 53700 | 2.02 ± 1.04% | 12.9 ± 4.25% | 27.1 ± 2.57% | 58.0 ± 2.83% | 2.4 |
| **45s** | 66873 | 8.53 ± 0.497% | 30.1 ± 0.833% | 46.7 ± 1.19% | 14.7 ± 0.376% | 1.68 | 74092 | 1.50 ± 0.76% | 9.58 ± 1.56% | 32.7± 2.45% | 56.2 ± 3.92% | 2.43 |
| **60s** | 66873 | 8.30 ± 2.89% | 32.8 ± 3.32% | 45.8 ± 6.92% | 13.1 ± 2.60% | 1.64 | 60321 | 0.66 ± 0.66% | 7.16 ± 2.22% | 21.3 ± 4.91% | 70.93 ± 7.00% | 2.61 |
| **90s** | 70959 | 10.8 ± 1.74% | 30.6 ± 1.64% | 46.6 ± 1.96% | 12.0 ± 1.32% | 1.60 | 76355 | 0 ± 0% | 6.16 ± 2.78% | 25.2 ± 2.42% | 68.7 ± 4.76% | 2.64 |
| **150s** | 145876 | 11.8 ± 3.18% | 30.0 ± 1.93% | 44.3 ± 3.20% | 13.9 ± 0.840% | 1.60 | 92790 | 0.24 ± 0.24% | 5.00 ± 1.44% | 19.8 ± 2.39% | 75.9 ± 3.96% | 2.7 |
| **24hr** | 54805 | 3.56 ± 2.22% | 16.7 ± 4.77% | 48.7 ± 1.89% | 31.1 ± 8.61% | 2.07 | 53591 | 0.17 ± 0.17% | 4.20 ± 1.09% | 19.61 ± 5.52% | 76.0 ± 6.47% | 2.7 |
